# Translation and Psychometric Properties of the Persian Version of the Organizational Learning Instrument–Development Stages (OLI-DS) Instrument in Hospital Units

**DOI:** 10.1155/jonm/3906448

**Published:** 2024-11-18

**Authors:** Alireza Mirzaei, Faranak Kazemi Darabadi, Sahar Havaskar, Azade Lotfi, Reza Nemati-Vakilabad

**Affiliations:** ^1^Department of Emergency Nursing, School of Nursing and Midwifery, Ardabil University of Medical Sciences, Ardabil, Iran; ^2^Department of Surgery, School of Medicine, Ardabil University of Medical Sciences, Ardabil, Iran; ^3^Department of Community Health Nursing, School of Nursing and Midwifery, Guilan University of Medical Sciences, Rasht, Iran; ^4^Department of Medical-Surgical Nursing, School of Nursing and Midwifery, Ardabil University of Medical Sciences, Ardabil, Iran

**Keywords:** nurse administrators, nurse managers, nursing, psychometrics, safety

## Abstract

**Aim:** The Organizational Learning Instrument–Development Stages (OLI-DS) measure hospital units' readiness to engage in organizational learning. This study aimed to translate and evaluate the psychometric properties of the Persian version of this instrument.

**Background:** Organizational learning is crucial for nurses as it enables them to continuously develop their knowledge, skills, and abilities, enhancing the quality of patient care. However, validated instruments are needed to assess organizational learning in Persian-speaking healthcare settings.

**Design:** The research was carried out using a cross-sectional design.

**Methods:** This research study involved 319 clinical nurses selected using convenience sampling. The OLI-DS tool, consisting of 35 items, underwent a forward–backward translation process to Persian before being utilized for a psychometric analysis. The face, content, and construct validity were used to evaluate the tool's effectiveness. The quality and accuracy of the measurements were determined by calculating internal consistency and stability reliability. The data were analyzed using SPSS and AMOS software.

**Results:** The intended meaning and clarity of the original English version were preserved in the Persian rendition of the OLI-DS instrument. The confirmatory factor analysis (CFA) further confirmed the consistency of the Persian version with the proposed four-factor model, indicating a good fit. The overall instrument had excellent values for Cronbach's alpha coefficient (*α* = 0.931), McDonald's omega (*ω* = 0.921), coefficient H (*H* = 0.979), and mean interitem correlation (*ρ* = 0.278). The convergent and discriminant validity of the four latent factors was found to be good. The stability of the overall OLI-DS instrument was ICC = 0.942 (95% CI, 0.902–0.955).

**Conclusion:** The Persian version of the OLI-DS has been validated to evaluate hospital units' readiness for organizational learning processes. It is a robust tool for assessing their preparedness to participate in and benefit from organizational learning initiatives.

**Implications for Nursing Management:** The robust psychometric validation of the Persian version of the OLI-DS instrument equips nursing administrators and leaders with a reliable tool. This tool can assess and monitor organizational learning and development in healthcare settings, empowering them to make informed decisions in critical nursing management activities.

## 1. Introduction

Healthcare organizations can experience significant growth and improvement in processes and services, leading to safe, high-quality care in a changing environment [1]. Organizational learning is a crucial driver that enhances actions by acquiring better knowledge, leading to improved overall performance [2]. Evidence suggests that an organization's learning capacity grows as it develops. Organizational learning is the active process of acquiring, creating, and combining knowledge to improve performance and build resources and competencies [3]. Healthcare managers should prioritize learning as an essential component of daily operations at all levels to ensure the organization's success [4]. Nurse leaders can improve organizational learning and intervention assessment by using a tool to measure hospital unit progress. This study aimed to translate and assess the reliability and validity of this tool.

### 1.1. Background

Healthcare organizations must implement new strategies to provide services effectively while collaborating with the environment and other organizations. One key strategy is prioritizing nurses' learning management in hospitals, which involves developing knowledge policies, techniques, and action plans; promoting continuous improvement; and establishing standard mental models [5]. Healthcare management experts recommend organizational learning as an effective method to improve the safety and quality of healthcare [6]. Lyman et al. conducted a study on organizational learning in high-performing hospital units. They identified four stages of growth in this process: (1) identity and ownership, (2) team and respect, (3) accountability and support, and (4) reliability and sustainability [7, 8]. In organizational learning, each stage has a unique focus. For example, in the identity and ownership stage, team members must establish a clear vision and take responsibility. A collaborative approach is essential in the team and respect stage, while performance expectations should be defined in the accountability and support stage. Continuous engagement helps teams adapt to change and reach reliability and sustainability [3]. Influential nurse leaders facilitate organizational learning by inspiring, motivating, and guiding their teams [9].

It is crucial for hospitals to continuously improve patient care and outcomes through organizational learning, open communication, collaboration, professional development, and provision of resources [10]. Organizational learning in healthcare has been linked to improved clinical outcomes, enhanced patient experience, better team performance, and greater financial sustainability [11]. Nurses play a vital role in preventing and treating diseases, making them essential to any health promotion team [12]. Hospitals can improve healthcare services by promoting organizational learning management among nurses [13]. Ultimately, society's health depends on nurses' professional growth, expertise, and dedication to provide high-quality medical and health services to those in need [14].

Two assessment instruments translated into Persian to measure organizational learning among Iranian nurses are not explicitly designed for this context [15, 16]. This lack of specificity is a significant problem because it hinders the accurate assessment of nurses' learning within the organization. For instance, the tool developed by Flores et al. to assess organizational learning at the unit level in healthcare is not exclusive to nursing; it is intended for broader use [17]. These instruments do not cater specifically to nursing. There is an urgent need for a nursing-specific organizational learning tool that addresses the unique aspects of organizational learning among nurses, such as team development and effective subprocesses. Developing this tool is crucial for enhancing healthcare services in Iran. The OLI-DS is reliable for assessing organizational learning in hospital units [3]. It identifies four stages, enabling managers to evaluate performance and improve outcomes, such as the quality of care and staff satisfaction. It has undergone rigorous testing and consistently demonstrates reliable results across different settings.

The authors emphasize the importance of implementing a measurement scale in Iran to assist hospital managers in improving team development and patient outcomes. Our research, which is essential for the progress of Iranian hospitals, focuses on validating a Persian-language OLI-DS. This tool can help Iranian nursing managers evaluate organizational learning levels and create strategies to enhance performance and patient outcomes. It enables managers to identify strengths and weaknesses and improve organizational learning for better performance and patient care outcomes, highlighting the significance of their role in the hospital's success.

## 2. Methods

### 2.1. Aim

The current study translated and evaluated the psychometric properties of the OLI-DS in Persian. The OLI-DS measures hospital units' capacity for organizational learning and aims to support learning in healthcare settings.

### 2.2. Design and Setting

This methodological study assessed the psychometric properties of the Persian version of the OLI-DS in hospitals. The research was carried out from September to December 2023. Convenience sampling was utilized as the sampling method. The study involved clinical nurses from five educational and therapeutic hospitals in Ardabil province, which is in the northwest region of Iran.

### 2.3. Participants

The study gathered data from 319 clinical nurses employed in affiliated educational-therapeutic hospitals of Ardabil University of Medical Sciences. The participants were chosen using a convenience sampling method. Participants with at least a bachelor's degree in nursing and more than 6 months of work experience were eligible for the study provided they agreed to participate willingly. The only reason for exclusion from the study was incomplete questionnaire responses.

### 2.4. Instrument

The initial part of the tool comprises a demographic questionnaire that covers aspects such as age, gender, marital status, education, experience, and the department in which the participant works. The second section of the instrument was the OLI-DS among clinical nurses, designed by Lyman and Ethington [3]. OLI-DS is an instrument consisting of 35 items and four subscales: identity and ownership (13 items), team and respect (6 items), accountability and support (10 items), and reliability and sustainability (6 items). The items are rated on a 4-point Likert scale (1 = *strongly disagree* to 4 = *strongly agree*). When analyzing data collected from the OLI-DS instrument, it is essential to focus on the unit level. The reason for using this instrument is that the survey requests the participants to provide details about the hospital unit they are employed in rather than their personal information. Therefore, if the scores on the survey are higher, it suggests that organizational learning is better.

### 2.5. Psychometric Evaluation of the OLI-DS Instrument

The researchers in this study got permission from the developer of OLI-DS, Dr. Bret Lyman, to use the instrument. The team translated the instrument into Persian, following the World Health Organization (WHO) guidelines to ensure accuracy. First, two translators specializing in nursing translated the original instrument into Persian. The team compared the Persian version to the English original, creating a standardized version. Afterwards, an expert translator who specialized in nursing but needed to become more familiar with the original version of the instrument translated the Persian version of OLI-DS into English. Later, the instrument developer examined the translation to confirm that it effectively communicated the main ideas and terminology. The feedback was used to create the final Persian version of OLI-DS. The researchers evaluated the final Persian version of OLI-DS for validity and reliability. We conducted a comprehensive testing process to validate the instrument with a carefully selected sample of Persian-speaking nurses. This rigorous evaluation was essential to confirm that the instrument consistently produced accurate results within this demographic.

We used qualitative and quantitative methods to assess the instrument's face validity. For the qualitative method, we purposely selected ten clinical nurses and administered the OLI-DS instrument to them. We used qualitative and quantitative approaches to assess the instrument's face validity. For the qualitative method, we purposely selected ten clinical nurses and administered the OLI-DS instrument to them. We inquired about their viewpoints regarding the items' importance through a qualitative method. We utilized their opinion to enhance the text and render it more comprehensible by eliminating vague expressions.

Clinical nurses were asked to rate each item on a five-point Likert scale, ranging from “*not important*” to “*quite important*,” to assess its suitability. The researchers analyzed the impact score of each item. The researchers studied to determine the importance and frequency of each item. They calculated the impact score for each item by multiplying its frequency by importance. Based on this, they identified the frequency of scores of four or 5five and determined the average score the nurses gave for each item. The researchers concluded that an impact score of 1.5 or higher was appropriate for each item based on their analysis [18].

The OLI-DS instrument underwent a process of calculating its item-level content validity index (I-CVI) and scale-level content validity index (S-CVI). Each item of the OLI-DS was evaluated for I-CVI by dividing the number of experts rating three or four by the total number of experts involved in the CVI assessment [19]. To obtain the S-CVI/Ave, the I-CVI values were averaged across all the items. The acceptable ranges for I-CVI and S-CVI/Ave are > 0.78 and > 0.90, respectively [19, 20]. Some critics argue that the CVI process does not consider the possibility of inflated values resulting from a chance agreement among experts [19]. To address this issue, we made a content validity index correction of the probability of chance agreement (*p*_*c*_) using the formula: *p*_*c*_=[*N*!/(*A*!(*A*!(*N* − *A*)!)] × 0.5N (where *N* = the number of experts and *A* = the number agreeing on good relevance). We then calculated the modified kappa (*k*^∗^=(I − CVI − *p*_*c*_)/(1 − *p*_*c*_)) for each item of the tool. The *k*^∗^ values were then assessed based on the following criteria: poor: *k*^∗^ values ≤ 0.39; fair: *k*^∗^ values = 0.40–0.59; good: *k*^∗^ values = 0.60–0.74; and excellent: *k*^∗^ values > 0.74) [19].

We ensured the accuracy of the assessment by analyzing whether too many individuals scored at either the highest or lowest level. If the percentage of participants who achieved the highest or lowest possible score exceeded 15%, this phenomenon is called a floor or ceiling effect. In such cases, the tool may lack questions that accurately represent the lowest or highest level of the measured characteristic. The issue at hand is that there is a concern that the tool may need to be more effectively measure all of the parameters it is designed to measure [21].

In this study, confirmatory factor analysis (CFA) was used to assess the construct validity of the OLI-DS scale, which was initially developed using exploratory factor analysis (EFA) [22]. CFA is more reliable than EFA in determining construct validity [23]. CFA is considered superior to EFA for determining construct validity. The parameters are estimated using maximum likelihood estimation (MLE). The fit indices such as *χ*^2^/df < 3, RMSEA < 0.08, GFI > 0.90, CFI > 0.90, TLI > 0.90, IFI > 0.90, and AGFI > 0.80 were used to assess the model's suitability [24, 25]. Factor loadings > 0.3 and T-values > 1.96 were considered acceptable and statistically significant [26].

Different opinions exist among researchers regarding the minimum sample size necessary for carrying out CFA. Some argue for a minimum of 100 participants, while others suggest a range between 100 and 400. A recent study recommends a minimum of 250 participants for accurate results [23, 26–31]. The study had an adequate number of participants, and thorough data collection was done to facilitate the performance of a CFA in line with the specified parameters. The OLI-DS scale comprised 35 items, with a sample of 10 participants assigned to each item, resulting in the selection of 350 clinical nurses through a convenience sampling approach. Following the exclusion of outliers and missing data, the response rate was 91%, yielding valid data from 319 clinical nurses, which was considered appropriate for further CFA analysis.

Convergent validity is attained in a study when the measured items possess a high degree of shared variance and are similar. On the other hand, discriminant validity is the term used to describe a situation where the measured items are unrelated or similar [26]. The OLI-DS tool was evaluated for its convergent and discriminant validity in this research, using different standards such as composite reliability (CR), average variance extracted (AVE), maximum shared squared variance (MSV), and average shared squared variance (ASV). Indicators for the latent variable are considered suitable when AVE values are high. For convergent validity, AVE values should be > 0.5, and CR values should be > 0.7 [26, 32–34]. This study used the heterotrait–monotrait (HTMT) correlation ratio to assess discriminant validity. According to this criterion, HTMT matrix values should be less than 0.9 to demonstrate discriminant validity [35].

An acceptable range was considered to be values greater than 0.7 for α, ω, and H [36–41]. An acceptable mean inter-item correlation ranges between 0.15 and 0.5 [42]. Correlations below 0.15 may indicate a weak relationship, while those above 0.5 may suggest redundancy. A coefficient of 0.4–0.5 is preferable for a valid measure [42].

Test–retest reliability was assessed using the intraclass correlation coefficient (ICC) with a two-way random model. Data from 40 clinical nurses, collected two weeks apart, aimed for an ICC value of 0.75 or higher [43].

### 2.6. Data Analysis

The data were examined for outliers using two methods. Univariate outliers were checked based on skewness and kurtosis values. Multivariate outliers were identified using the Mahalanobis squared distance method with a significance level of *p* < 0.001. The normality of multivariate variables was tested using Mardia's coefficient [44]. The data were analyzed using IBM SPSS Statistics for Windows software, version 26.0 (IBM Corp., Armonk, New York, United States of America). CFA was performed using AMOS, version 24.0 (IBM Corp., Armonk, New York, United States of America). The statistical significance level was set at *p* < 0.05.

### 2.7. Ethical Considerations

The Ardabil University of Medical Sciences Ethics Committee approved this study under the code IR.ARUMS.REC.1402.062. The translation process was only authorized after the tool developer received written consent via email. Before agreeing to participate in the research project, all participants were informed of the study's objective. The current research adhered to the guidelines stipulated in the Declaration of Helsinki. The participants willingly provided written consent without any pressure or intimidation. No coercion or threats were involved in obtaining their permission, and participants could withdraw at any time.

## 3. Results

### 3.1. Participant Characteristics

Three hundred and nineteen clinical nurses participated in this study, averaging 30.96 ± 3.50 years. Most participants were female (*n* = 174, 54.5%) and married (*n* = 180, 56.4%).  Table 1 summarizes the participants' baseline characteristics.

### 3.2. Translation Phase

Three professionals independently evaluated the final version of the OLI-DS and compared it with the initial version in English. The assessment outcomes demonstrated that the Persian version of the OLI-DS sustained the planned significance of the initial English version and was lucid, precise, and straightforward to comprehend.

### 3.3. Face and Content Validity

During the face validity process of the translated version of the OLI-DS, we were given feedback from clinical nurses. Their feedback helped us to identify areas of improvement, such as difficulty, relevancy, and ambiguity. We made minor revisions based on their suggestions. As shown in Table 2, all items in the translated version received a score of 1.5 or higher, ranging from 2.5 to 4.7. We decided to keep all items in the translated version of OLI-DS for further stages, as we believed every item was essential for the target population. Therefore, no items were removed at this stage.

Table 2 shows the content validity assessment findings for the OLI-DS and its items. The S-CVI/Ave score was 0.954, signifying that the entire OLI-DS is relevant at an acceptable level. Furthermore, almost all individual items had exceptional content validity (I-CVI > 0.78, *k*^∗^ > 0.74).  Table 3 shows that none of the OLI-DS instruments or the four dimensions of the Persian version of the OLI-DS demonstrated a floor or ceiling effect (< 15%).

### 3.4. Descriptive Statistics of the 35-Item OLI-DS

Table 3 displays the descriptive statistics of the Persian version of the 35-item OLI-DS. The instrument's average score was 3.01 (0.46). Subscales' average scores were identity and ownership 2.94 (0.68), team and respect 3.01 (0.67), accountability and support 3.18 (0.65), and reliability and sustainability 3.07 (0.66). The results suggest that participants generally held positive views of all the items.

### 3.5. Construct Validity

**Figure 1:**
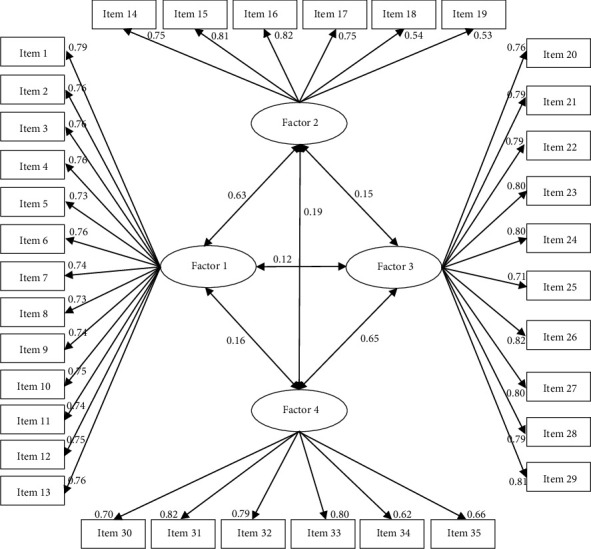
The confirmatory factor analysis model of the OLI-DS (*n* = 319).

We used the CFA approach to assess the construct validity of the OLI-DS tool. The CFA revealed that all factors had factor loadings greater than 0.3, and the connections between the dimensions and the items were statistically significant. The proposed model and its concepts were considered acceptable based on several goodness-of-fit indices, including *χ*^2^ = 1213.37, df = 571, *p* < 0.001, *χ*^2^/df = 2.125, RMSEA = 0.055, GFI = 0.919, CFI = 0.927, TLI = 0.921, IFI = 0.927, and AGFI = 0.863 (Table 4 and Figure 1).

### 3.6. Convergent and Discriminant Validity

All factors showed strong convergent validity, supported by the values of CR and AVE, which exceed the recommended benchmarks of 0.7 and 0.5, respectively. In addition, the value of CR was greater than that of AVE. Discriminant validity of the latent factors was also confirmed, as MSV and ASV values were lower than AVE values. Furthermore, the HTMT matrix analysis demonstrated that all values are less than 0.9, further supporting the discriminant validity among the factors (Table 5).

### 3.7. Reliability

The internal consistency of the OLI-DS structure, which consisted of 35 items, was found to be excellent (*α* = 0.931, *ω* = 0.921, coefficient *H* = 0.979, and *ρ* = 0.278). Factor one (identity and ownership) comprised of 13 items (*α* = 0.914, *ω* = 0.948, coefficient *H* = 0.945, and *ρ* = 0.464), factor two (team and respect) comprised of six items (*α* = 0.851, *ω* = 0.863, coefficient *H* = 0.880, and *ρ* = 0.487), factor three (accountability and support) comprised of 10 items (*α* = 0.902, *ω* = 0.947, coefficient *H* = 0.943, and *ρ* = 0.479), and factor four (reliability and sustainability) comprised of six items (*α* = 0.839, *ω* = 0.880, coefficient *H* = 0.886, and *ρ* = 0.464). The internal consistency of all these factors was found to be excellent, as shown in Table 6.

The stability of the OLI-DS instrument was assessed through a test–retest process. The results indicated overall stability of ICC = 0.942, with subcategories such as identity and ownership (ICC = 0.951), team and respect (ICC = 0.869), accountability and support (ICC = 0.950), and reliability and sustainability (ICC = 0.895) (Table 6).

### 3.8. Production of the Final Model

After assessing its validity and reliability, OLI-DS's Persian version, which consists of 35 items, was completed. These items were divided into four dimensions: “identity and ownership” (13 items), “team and respect” (six items), “accountability and support” (ten items), and “reliability and sustainability” (six items).

## 4. Discussion

We studied the OLI-DS tool's psychometric properties for evaluating hospital organizational learning. The study involved 319 clinical nurses from hospitals affiliated with Ardabil University of Medical Sciences. The OLI-DS scale assesses hospital units' readiness for various organizational situations, addressing the need for better preparation as indicated by the previous research [7, 8]. The study confirmed the validity and reliability of the Persian version of the OLI-DS tool in assessing organizational learning in Iranian healthcare settings.

The OLI-DS scale assesses organizational learning with 35 items divided into four subscales: identity and ownership (13 items), team and respect (six items), accountability and support (10 items), and reliability and sustainability (six items). It identifies areas for improvement in hospital units, guiding them towards becoming learning organizations. According to Lyman et al. [3, 7, 8], hospital units evolve into learning organizations through distinct developmental phases. The first involves creating a clear identity and sense of ownership. The second phase focuses on a team-oriented approach and promotes respectful behavior. In the third phase, units establish rules for accountability and implement measures to achieve and sustain desired performance levels through systems-based processes.

The precision of a measuring tool affects its accuracy [45]. To determine the content validity of the OLI-DS instrument, we engaged ten independent experts in a collaborative evaluation of how well the scale measures its intended purpose [46]. The strength of this study lies in the input from experts not affiliated with the research team, ensuring a collaborative and inclusive process. We used qualitative and quantitative methods to assess the face validity of the instrument. Clinical nurses were the target group for the face validity assessment, and their input was crucial [47]. Based on feedback from ten clinical nurses, some items in the scale were partially rewritten, further highlighting the collaborative nature of the research. All included items were deemed acceptable, suggesting that the scale's form and content are suitable.

The main goal of the CFA was to assess the validity of the OLI-DS assessment tool, which is meant to measure various aspects of an object [48]. The research findings confirmed that the original OLI-DS instrument had a consistent four-factor structure. The results showed that the model fit was good, with all indicators meeting the required standards and aligning with the original instrument [3]. The researchers also discovered that the OLI-DS tool is well-suited for future studies on organizational learning in educational and therapeutic hospitals affiliated with Ardabil University of Medical Sciences. In addition, the results demonstrated that the OLI-DS instrument exhibits both convergent and discriminant validity, indicating its effectiveness.

It is essential for a scale to consistently measure the intended construct, as determined by the reliability analysis [45]. The reliability of the OLI-DS instrument was assessed using Cronbach's alpha (*α* = 0.931), McDonald's omega (*ω* = 0.921), coefficient H (0.979), and mean interitem correlation (*ρ* = 0.278), indicating strong reliability comparable to the original version, with Omega coefficients ranging from 0.981 to 0.993 for its four factors. The scale's internal consistency is suitable, with items showing sufficient correlation, suggesting it effectively measures closely related aspects of organizational learning. Comprising 35 items rated on a 4-point Likert scale, higher scores reflect more excellent organizational learning. In addition, the OLI-DS can enhance learning by assessing team development, selecting strategies, and tracking progress, with data analysis conducted at the unit level to facilitate discussion and improvement.

The Persian version of the OLI-DS instrument underwent a comprehensive psychometric evaluation. However, the study had limitations, such as using a convenience sampling method that may have reduced external validity and limited the generalizability of the findings. In addition, concurrent validity testing and other forms of construct validity were not included. Nevertheless, the study's credibility was bolstered by conducting a CFA on 319 clinical nurses from Iran, surpassing the recommended sample size with acceptable model fitness and goodness-of-fit indices. Future research should seek input from diverse healthcare team members to understand organizational learning in healthcare settings comprehensively. Longitudinal studies are also necessary to explore the proposed developmental stages' model and their impact on overall organizational learning and significant outcomes.

### 4.1. Implications for Nursing Management

The Persian version of the OLI-DS instrument has been validated, providing nursing administrators with a reliable tool for evaluating organizational learning and development in healthcare. This tool facilitates workforce planning, professional development, quality improvement, change management, and benchmarking against standards [49]. Nursing managers can use the insights gained to identify areas for growth and implement strategies to promote a culture of learning and enhance the quality of healthcare delivery.

## 5. Conclusions

Organizational learning is crucial for improving key outcomes within hospital units. The recent research has shown that the OLI for Hospital Units (OLI-DS) is an effective tool for evaluating the progress of hospital units in terms of organizational learning. Nurse administrators and managers can use the OLI-DS to assess and improve team development within their respective units. However, further research is needed to understand how hospital units progress through different stages of development, the factors that influence this progression, and the implications of this evolution on overall organizational learning and other vital outcomes. This ongoing investigation brings hope for continual advancement and improvement in healthcare settings.

## Figures and Tables

**Table 1:** Sociodemographic information of the participants (*n* = 319).

Variables	Categories	Mean ± SD	
Age (year)		30.96 ± 3.50	
Working experience (year)		8.82 ± 3.24	

		**No.**	**Percentage**

Gender	Male	145	45.5
	Female	174	54.5

Marital status	Single	139	43.6
	Married	180	56.4

Education level	Bachelor's degree	230	72.1
	Master's degree	89	27.9

Working department	Medical	92	28.8
	Surgical	68	21.3
	Emergency	32	10.1
	ICU	34	10.7
	Pediatric	23	7.2
	Other	70	21.9

**Table 2:** The results for the face and content validity of the OLI-DS (*n* = 319).

Item	Impact score	A	I-CVI	*p*_*c*_	*k* ^∗^
1	4.7	10	1	0.001	1
2	4.7	10	1	0.001	1
3	3.8	9	0.9	0.01	0.89
4	2.5	8	0.8	0.044	0.79
5	3.2	10	1	0.001	1
6	3.7	10	1	0.001	1
7	4.3	10	1	0.001	1
8	2.5	8	0.8	0.044	0.79
9	3.4	9	0.9	0.01	0.89
10	2.7	8	0.8	0.044	0.79
11	4.7	10	1	0.001	1
12	3.8	10	1	0.001	1
13	3.4	10	1	0.001	1
14	3.7	10	1	0.001	1
15	2.7	10	1	0.001	1
16	3.8	10	1	0.001	1
17	4.7	9	0.9	0.01	0.89
18	2.5	10	1	0.001	1
19	2.7	10	1	0.001	1
20	3.2	10	1	0.001	1
21	2.7	10	1	0.001	1
22	4.3	10	1	0.001	1
23	3.7	10	1	0.001	1
24	3.2	8	0.8	0.044	0.79
25	3.2	10	1	0.001	1
26	3.4	9	0.9	0.01	0.89
27	2.5	10	1	0.001	1
28	3.2	10	1	0.001	1
29	2.7	10	1	0.001	1
30	2.7	8	0.8	0.044	0.79
31	4.7	10	1	0.001	1
32	3.8	10	1	0.001	1
33	2.7	9	0.9	0.01	0.89
34	3.4	10	1	0.001	1
35	3.4	9	0.9	0.01	0.89

*Note:* A, number agreeing on good relevance; *p*_*c*_, probability of a chance occurrence; *k*^∗^, kappa-designating agreement on relevance.

Abbreviation: I-CVI, item content validity index.

**Table 3:** Descriptive statistics and floor and ceiling effects of the 35-item OLI-DS (*n* = 319).

Dimensions	No. of item	Possible range	Mean ± SD	Skewness	Kurtosis	Floor effect (%)	Ceiling effect (%)
Identity and ownership	13	1–4	2.94 ± 0.68	−0.243	−0.785	3 (0.9%)	22 (6.9%)
Team and respect	6	1–4	3.01 ± 0.67	−0.362	−0.454	4 (1.25%)	21 (6.6%)
Accountability and support	10	1–4	3.18 ± 0.65	−0.767	−0.617	7 (2.9%)	23 (7.2%)
Reliability and sustainability	6	1–4	3.07 ± 0.66	−0.693	−0.541	7 (2.9%)	36 (11.3%)
Total	35	1–4	3.01 ± 0.46	−0.130	−0.623	1 (0.3%)	2 (0.6%)

**Table 4:** Goodness of fit statistics for CFA models of the OLI-DS (*n* = 319).

Indices	*χ* ^2^	df	*p *value	*χ* ^2^/df	RMSEA	GFI	CFI	TLI	IFI	AGFI
CFA model	1213.37	571	0.001	2.125	0.055	0.919	0.927	0.921	0.927	0.863
Acceptable values	—	—	> 0.05	< 3	< 0.08	> 0.90	> 0.90	> 0.90	> 0.90	> 0.80

*Note:χ* ^2^/df, ratio of chi-square to its degree of freedom.

Abbreviations: AGFI, adjusted goodness of fit index; CFI, comparative fit index; GFI, goodness of fit index; IFI, incremental fit index; RMSEA, root mean square error of approximation; TLI, Tucker–Lewis index.

**Table 5:** Indices of the convergent and discriminant validity of the OLI-DS (*n* = 319).

Dimensions	CR	AVE	MSV	ASV	HTMT			
					Identity and ownership	Team and respect	Accountability and support	Reliability and sustainability
Identity and ownership	0.944	0.565	0.397	0.145	—			
Team and respect	0.856	0.504	0.397	0.151	0.611	—		
Accountability and support	0.953	0.620	0.422	0.153	0.123	0.297	—	
Reliability and sustainability	0.875	0.541	0.422	0.161	0.367	0.368	0.794	—

Abbreviations: ASV, average shared squared variance; AVE, average variance extracted; CR, composite reliability; HTMT, heterotrait–monotrait ratio of correlation; MSV, maximum shared squared variance.

**Table 6:** Internal consistency and stability for the OLI-DS (*n* = 319).

Dimensions	*α*	*ω*	H	*ρ*	ICC (95% CI)
Identity and ownership	0.914	0.948	0.945	0.464	0.951 (0.908–0.963)
Team and respect	0.851	0.863	0.880	0.487	0.869 (0.790–0.921)
Accountability and support	0.902	0.947	0.943	0.479	0.950 (0.912–0.978)
Reliability and sustainability	0.839	0.880	0.886	0.464	0.895 (0.789–0.906)
Total	0.931	0.921	0.979	0.278	0.942 (0.902–0.955)

*Note: α*, Cronbach's alpha; *ω*, McDonald's omega coefficient; H, coefficient H; *ρ*, mean interitem correlation.

Abbreviations: CI, confidence interval; ICC, intraclass correlation coefficient.

## Data Availability

The data that support the findings of this study are available from the corresponding author upon request.
